# Early-Onset Takayasu Arteritis in Childhood: A Case Report

**DOI:** 10.7759/cureus.53885

**Published:** 2024-02-08

**Authors:** Mridu Bahal, Gaurav Kumar, Shailaja Mane, Sanjay Chavan, Aryan Gupta

**Affiliations:** 1 Pediatrics, Dr. D. Y. Patil Medical College, Hospital and Research Centre, Dr. D. Y. Patil Vidyapeeth (Deemed to be University), Pune, IND

**Keywords:** takayasu arteritis, systemic inflammation, childhood onset, end organ ischemia, large vessel vasculitis

## Abstract

Takayasu arteritis is a chronic, idiopathic, inflammatory disease mainly affecting medium and large vessels with a significant rate of morbidity and mortality. The vessels most frequently affected are the aorta and its branches; branches originating from the aortic arch include right brachiocephalic trunk and its branches, left common carotid artery, left subclavian artery, coronary arteries from the ascending aorta, celiac trunk, right and left renal arteries, superior and inferior mesenteric arteries from the descending aorta, and right and left iliofemoral arteries. Local and systemic inflammation along with end organ ischemia is attributed to severe clinical manifestations associated with this condition.

Although Takayasu arteritis is more commonly diagnosed in adults, this study highlights the unusual occurrence of childhood-onset Takayasu arteritis (TAK), presenting a unique set of diagnostic challenges. We present a case of a seven-year-old female patient who manifested atypical symptoms, such as absent pulses and malignant hypertension at an early age, leading to a delayed diagnosis. The patient's clinical course, including diagnostic workup and imaging studies such as CT or MR angiography, is thoroughly discussed. This study emphasizes the importance of recognizing the subtleties of Takayasu arteritis in children. The disease may initially masquerade as other common conditions, such as peripheral arterial disease, coarctation of aorta, renal artery stenosis, chronic renal disease, and increased intracranial pressure, thereby hindering timely diagnosis and appropriate intervention.

This case underscores the importance of considering Takayasu arteritis as a differential diagnosis in children, presenting with unexplained constitutional symptoms or signs of systemic vasculitis, emphasizing the need for multidisciplinary collaboration and tailored therapeutic intervention to optimize the outcome in this rare and potentially debilitating condition.

## Introduction

Takayasu arteritis was first reported in 1908 by Dr. Mikito Takayasu at Kanazawa University in a 22-year-old female [1]. This case was first reported as prominent abnormalities in the retinal vessels of the patient.

Takayasu arteritis is a chronic and most common form of large-vessel vasculitis in children [2]. This condition is associated with aorta and its major branches, mainly aortic arch and its direct branches, renal arteries, and less commonly pulmonary arteries showing a characteristic granulomatous inflammation, and the infiltrates majorly constitute macrophages and lymphoid cells [3]. Increased expression of Th1 and Th17 cells has been reported to be correlated with inflammation in Takayasu arteritis [4]. Aneurysms and dissections are also frequently seen as a result of vessel wall inflammation, which causes thickening, stenosis, and thrombus formation [5]. The adventitia, outer section of the tunica media, and vasa vasorum are typically involved in the inflammatory process, which damages the artery wall by causing laminar necrosis and elastic fiber fragmentation [2]. Fibrosis and arterial remodeling eventually take over. The diagnosis, supported by laboratory results, is based on clinical criteria and angiographic abnormalities. HLA-B52 allele is reported to be a characteristic allele in Takayasu arteritis across multiple ethnicities [6]. Although being a rare entity in pediatric population, especially in India, Takayasu arteritis is a potentially life-threatening disease in children, likely with a prolonged subclinical course leading to late presentation, diagnosis of Takayasu arteritis (TAK) should be considered if clinical features are supported by radiological evidence for the disease. Early diagnosis and prompt intervention can prevent life-threatening complications.

## Case presentation

A female patient in her middle childhood presented to the emergency room with frequent episodes of headache which were associated with light-headedness and vomiting for the past 10 days. On the day of admission, she developed sudden onset shortness of breath not associated with fever, cough, cold, stridor, hemoptysis, chest pain, pedal edema, and orthopnea. She also expressed concern about myalgia, decreased appetite, and weariness. Second order born to a non-consanguineous marriage via normal vaginal delivery with no history of NICU admission.

The patient was brought to the emergency room where her oxygen saturation was 70-75% on room air, with intercostal retractions present. Physical examination showed continuous hypertensive readings >99P with tachycardia and tachypnea. Her body temperature was 98.6°F and her heart and respiratory rates were 176 beats/min and 57 breaths/min respectively, with weak upper extremity pulses. Examination of chest and precordial area showed intercostal retractions and gallop rhythm. In the emergency room, she was taken on oxygen support and shifted to the pediatric intensive care unit (PICU) for further management. Hemogram and blood culture, gastric aspirate for acid-fast bacilli and cartridge-based nucleic acid amplification test (CBNAAT), serology, antistreptolysin O (ASO) titer, and urinalysis were negative. Blood investigation along with blood culture are presented in Table 1. Gastric aspirate for acid-fast bacilli and CBNAAT, serology, ASO titer, and urinalysis were negative.

**Table 1:** Hemogram of the patient.

Parameter	Value	Normal range
Hemoglobin	9.5 mg/dL	11.8-14.7 mg/dL
C-reactive protein	24.5 mg/dL	<5 mg/dL
D-dimer	32545 ng/mL	0-500 ng/mL
Pro-brain natriuretic peptide	29229.8 pg/nL	<125 pg/nL
Covid IgG antibody	Positive (2855.7 IU/mL)	Negative
Erythrocyte sedimentation rate (ESR)	58 mm/h	<15 mm/h

Fundoscopy was done to rule out hypertensive retinal changes which came out to be within normal limits. Ultrasonography of abdomen and pelvis was done which showed suspicious narrowing of aorta at and beyond origin of superior mesenteric artery and bilateral pleural effusion with subsegmental collapse of underlying lung parenchyma. The suprarenal diameter of aorta was 1.4 cm and renal and infrarenal diameters of aorta were 0.5-0.6 cm. Renal artery Doppler showed a resistive index to be borderline raised in bilateral renal artery origin. Two-dimensional echocardiography was suggestive of reduced ejection fraction (30%) with global dilated dysfunctional left ventricle, moderate mitral regurgitation (MR), and mild tricuspid regurgitation (TR) with normal valves.

**Figure 1:**
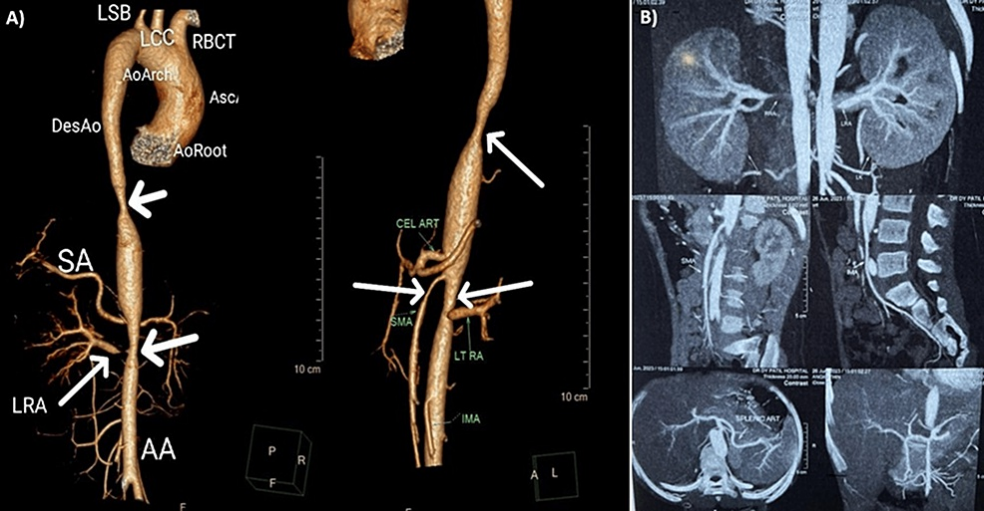
Radiology images confirming the diagnosis. CT aortogram showing (A) multiple segmental vascular stenosis with periaortic enhancing soft tissue and (B) wall thickening in the abdominal aorta, lower thoracic aorta, origin of bilateral renal arteries, right subclavian artery, and right axillary artery. LSB: left subclavian artery; LCC: left common carotid artery; RBCT: right brachiocephalic trunk (also known as innominate artery); DesAo: descending aorta; ASC: ascending aorta; AoRoot: aortic root; SA: subclavian artery; LRA: left renal artery; AA: abdominal aorta; CEL ART: celiac artery; SAM: superior mesenteric artery; IMA: inferior mesenteric artery

The patient was further evaluated via CT aortogram which showed multiple segmental vascular stenosis with periaortic enhancing soft tissue/wall thickening in abdominal aorta, lower thoracic aorta, origin of bilateral renal arteries, right subclavian artery, and right axillary artery which was suggestive of TAK arteritis, cardiomegaly with left atrial enlargement, and left ventricular dilatation with concentric hypertrophy. Minimal pericardial effusion and early changes in pulmonary venous hypertension were seen (Figures 1A, 1B).

Initially patient required oxygen support which was upgraded to a high-flow nasal cannula (HFNC) in the intensive care unit, over the course of four to five days, she was weaned off oxygen support gradually. Hypertension was aggressively managed, initially with oral amlodipine and IV furosemide which later was converted to continuous IV infusion of furosemide along with IV sodium nitroprusside infusion under careful monitoring, gradual infusions were weaned off over the next two days and stopped. Further hypertension was managed with oral amlodipine and oral spironolactone. To control the cardiac failure and improve cardiac output, IV milrinone and IV levosimendan infusions were added, which later were weaned off and stopped over two days and switched to oral carvedilol.

Once the diagnosis of TAK arteritis was confirmed, the patient was initially treated with IV cyclophosphamide (750 mg/m^2^) and IV methylprednisolone pulse therapy (30 mg/kg/day) for five days which was later converted to oral prednisolone (2 mg/kg/day) for three weeks.

After eight days of ICU stay with aggressive management, significant clinical improvement was noted along with stabilization of blood pressure readings. Patient was shifted out of the ICU unit on oral antihypertensive, oral steroids, and oral carvedilol. She was discharged after 13 days of hospital stay on oral hypertensive (amlodipine and prazosin), oral carvedilol, and oral prednisolone with left ventricular ejection fraction (LVEF) of 45-50%. She is currently under regular follow-up receiving IV cyclophosphamide on a daycare basis to maintain remission, steroids were stopped gradually after the completion of three-week course.

## Discussion

Takayasu arteritis incidences in childhood are exceedingly rare [2]. In young Asian females, Takayasu arteritis is reportedly a common cause of renovascular hypertension often associated with renal artery stenosis [7]. It is also associated with renal artery variations and micro-kidney [8]. Up to 25% of patients have been found to have aortic regurgitation and heart failure, with the average age of 24.0±8.8 years at onset of symptoms and 28.3±9.9 years at the time of diagnosis [9,10]. In 10-20% of these patients, acute ischemic strokes have been reported [11]. Vasculitic involvement or a previous embolization into the vessel in Takayasu arteritis could result in intracranial stenosis. An alternate source of embolism could be the involvement of proximal arteries and cardiac valves [12].

A common clinical mode of disease presentation in our patient was shortness of breath and hypertension, together with non-specific symptoms (vomiting, headache, myalgia). This condition is also called “pulseless disease,” due to the absence of peripheral pulses caused by vascular obstruction; however, it was present in our patient [7,13]. There is an unclear association between Takayasu arteritis and TB. Takayasu arteritis patients diagnosed with active tuberculosis (TB) account for about 20%. Our patient was not having active TB. The diagnostic criteria used in this study (developed by the Ankara conference) show 100% sensitivity and 99.9% specificity [14]. The criteria include the following standards: angiographic abnormalities must be proven by imaging studies. It should be supported by one of the following criteria: weak or absent peripheral pulses, difference in four limb systolic blood pressure measurements of more than 10 mmHg difference in any limb, bruits over large arteries, hypertension, and raised acute phase reactant. The patient at the time of diagnosis met every criterion mentioned above.

**Table 2:** Angiographic classification of Takayasu arteritis.

Type	Vessel involvement
Type I	Branches from the aortic arch
Type IIa	Ascending aorta, aortic arch, and its branches
Type IIb	Ascending aorta, aortic arch and its branches, and thoracic descending aorta
Type III	Thoracic descending aorta, abdominal aorta, and/or renal arteries
Type IV	Abdominal aorta and/or renal arteries
Type V	Combined features of types IIb and IV

According to the above-mentioned classification in Table 2 [15], our patient is classified as type V, which showed multiple segmental vascular stenosis with periaortic enhancing soft tissue/wall thickening in abdominal aorta, lower thoracic aorta, origin of bilateral renal arteries, right subclavian artery, and right axillary artery [16]. Treatment of Takayasu arteritis includes glucocorticoid as the first line of management; however, due to a relapse rate of 46-84% use of second-line immune suppressants to maintain increasing remission [15,16]. The patient was initially put on steroids and cyclophosphamide was added to achieve and maintain remission. This helped in the remission of both clinical symptoms and acute phase reactants, and therefore the patient was continued on steroids. Cyclophosphamide and antihypertensive medications were added to control this patient's blood pressure.

## Conclusions

Takayasu arteritis being a rare entity, particularly in young subset of patients, can be difficult to diagnose and treat for general pediatricians and specialists as well. Thorough clinical examination with diagnostic workup, especially CT or MR angiography, should be considered in children with hypertension, signs of systemic inflammation, and vascular involvement. Early diagnosis with prompt intervention leads to satisfactory outcomes with reduction in morbidity, mortality, and life-threatening complications. Although observation based on a large patient sample size should be considered; however, the low incidence of the disease is a limiting factor.
